# Intravenous Thrombolysis Before Thrombectomy Improves Functional Outcome After Stroke Independent of Reperfusion Grade

**DOI:** 10.1161/JAHA.123.031854

**Published:** 2024-03-08

**Authors:** Annahita Sedghi, Daniel P. O. Kaiser, Ani Cuberi, Sonja Schreckenbauer, Claudia Wojciechowski, Ingeborg Friehs, Heinz Reichmann, Jessica Barlinn, Kristian Barlinn, Volker Puetz, Timo Siepmann

**Affiliations:** ^1^ Dresden Neurovascular Center, Department of Neurology, Medical Faculty and University Hospital Carl Gustav Carus Dresden University of Technology Dresden Germany; ^2^ Division of Health Care Sciences Dresden International University Dresden Germany; ^3^ Dresden Neurovascular Center, Institute of Neuroradiology, Medical Faculty and University Hospital Carl Gustav Carus, Dresden University of Technology Dresden Germany; ^4^ Institute of Radiology, Medical Faculty and University Hospital Carl Gustav Carus, Dresden University of Technology Dresden Germany; ^5^ Department of Cardiac Surgery Boston Children’s Hospital, Harvard Medical School Boston MA USA

**Keywords:** acute stroke, ischemia, outcome, recanalization, recombinant tissue‐type plasminogen activator, Cerebrovascular Disease/Stroke, Ischemic Stroke, Treatment, Revascularization

## Abstract

**Background:**

We studied the association of bridging intravenous thrombolysis (IVT) before thrombectomy for anterior circulation large‐vessel occlusion and functional outcome and scrutinized its dependence on grade of reperfusion and distal thrombus migration.

**Methods and Results:**

We included consecutive patients with anterior circulation large‐vessel occlusion from our prospective registry of thrombectomy‐eligible patients treated from January 1, 2017 to January 1, 2023 at a tertiary stroke center in Germany in this retrospective cohort study. To evaluate the association of bridging IVT and functional outcome quantified via modified Rankin Scale score at 90 days we used multivariable logistic and lasso regression including interaction terms with grade of reperfusion quantified via modified Thrombolysis in Cerebral Infarction (mTICI) scale and distal thrombus migration adjusted for demographic and cardiovascular risk profiles, clinical and imaging stroke characteristics, onset‐to‐recanalization time and distal thrombus migration. We performed sensitivity analysis using propensity score matching. In our study population of 1000 thrombectomy‐eligible patients (513 women; median age, 77 years [interquartile range, 67–84]), IVT emerged as a predictor of favorable functional outcome (modified Rankin Scale score, 0–2) independent of modified mTICI score (adjusted odds ratio, 0.49 [95% CI, 0.32–0.75]; *P*=0.001). In those who underwent thrombectomy (n=812), the association of IVT and favorable functional outcome was reproduced (adjusted odds ratio, 0.49 [95% CI, 0.31–0.74]; *P*=0.001) and was further confirmed on propensity score analysis, where IVT led to a 0.35‐point decrease in 90‐day modified Rankin Scale score (ß=–0.35 [95 CI%, −0.68 to 0.01]; *P*=0.04). The additive benefit of IVT remained independent of modified mTICI score (ß=–1.79 [95% CI, −3.43 to –0.15]; *P*=0.03) and distal thrombus migration (ß=–0.41 [95% CI, −0.69 to –0.13]; *P*=0.004) on interaction analysis. Consequently, IVT showed an additive association with functional outcome in the subpopulation of patients undergoing thrombectomy who achieved successful reperfusion (mTICI ≥2b; ß=–0.46 [95% CI, −0.74 to –0.17]; *P*=0.002) and remained beneficial in those with unsuccessful reperfusion (mTICI ≤2a; ß=–0.47 [95% CI, −0.96 to 0.01]; *P*=0.05).

**Conclusions:**

In thrombectomy‐eligible patients with anterior circulation large‐vessel occlusion, IVT improves functional outcome independent of grade of reperfusion and distal thrombus migration.

**Clinical Perspective**

**What Is New?**

- Bridging intravenous thrombolysis before thrombectomy for anterior circulation stroke due to large‐vessel occlusion improves functional outcome compared with thrombectomy alone to an extent that cannot be explained solely by the instantaneous impact of improved postinterventional reperfusion.
- The beneficial albeit small association of bridging intravenous thrombolysis and functional outcome is independent of thrombus migration from a proximal to distal location on repeated vessel imaging and is also seen in the subgroup of patients with unsuccessful reperfusion following thrombectomy.

**What Are the Clinical Implications?**

- Bridging intravenous thrombolysis should not be withheld in patients with large‐vessel anterior circulation stroke who have an established indication for thrombectomy.

**Nonstandard Abbreviations and Acronyms**
acLVO: anterior circulation large vessel occlusion
ASPECTS: Alberta Stroke Program Early Computed Tomography Score
ICA: internal carotid artery
IVT: intravenous thrombolysis
MCA: middle cerebral artery
mRS: modified Rankin Scale
mTICI: modified Thrombolysis in Cerebral Infarction
NASCET: North American Symptomatic Carotid Endarterectomy Trial
NIHSS: National Institute of Health Stroke Scale
TOAST: Trial of Org 10 172 in Acute Stroke Treatment

Intravenous thrombolysis (IVT) and thrombectomy improve clinical outcome of acute ischemic stroke caused by cerebral anterior circulation large‐vessel occlusion (acLVO) in a highly time‐dependent fashion with a rapid decline of efficacy with extending time from onset of symptoms.^1^, ^2^ Several randomized controlled trials tested whether bridging IVT before thrombectomy has an additive beneficial effect on clinical outcome. These studies yielded conflicting results. While the European Multicenter Randomized Clinical Trial of Endovascular Treatment for Acute Ischemic Stroke in the Netherlands (MR CLEAN NO IV) trial and the Direct Mechanical Thrombectomy in Acute LVO Stroke (Japanese SKIP) study failed to demonstrate noninferiority of direct thrombectomy versus bridging IVT and thrombectomy, the Chinese studies Direct Intra‐Arterial Thrombectomy in Order to Revascularize AIS Patients With Large Vessel Occlusion Efficiently in Chinese Tertiary Hospitals: A Multicenter Randomized Clinical Trial (DIRECT‐MT) and Direct Endovascular Treatment Versus Standard Bridging Therapy in Large Artery Anterior Circulation Stroke (DEVT) were able to show noninferiority but were limited by wide margins.^3^, ^4^, ^5^, ^6^ The latter observation was recently supported by a meta‐analysis of clinical trials and observational studies that synthesized data from 36 123 patients and found slightly improved functional outcome and reperfusion rates in patients undergoing thrombectomy who also received bridging IVT.^7^ Moreover, the open‐label, blinded–end point, randomized trials Solitaire With the Intention For Thrombectomy Plus Intravenous t‐PA Versus DIRECT Solitaire Stent‐retriever Thrombectomy in Acute Anterior Circulation Stroke (SWIFT‐DIRECT) and a Randomized Controlled Trial of DIRECT Endovascular Clot Retrieval Versus Standard Bridging Thrombolysis With Endovascular Clot Retrieval (DIRECT‐SAFE) failed to show noninferiority of omitting bridging IVT before thrombectomy and even found effect directions in favor of bridging IVT.^8^, ^9^ Consequentially, the question of whether bridging IVT adds value to thrombectomy beyond noninferiority continues to be a topic of exploration. A recent retrospective cohort study in 746 patients with acLVO who underwent thrombectomy but did not achieve successful reperfusion found improved functional outcome after 90 days possibly mediated by improved cerebral macrocirculation and microcirculation.^10^ Here, we aimed to assess if IVT in thrombectomy‐eligible patients with acLVO has a beneficial association with functional outcome beyond an extent that can be explained by improvement of reperfusion as captured by the modified Thrombolysis in Cerebral Infarction (mTICI) scale.

## Methods

The data that support the findings of this study are available from the corresponding author upon reasonable request.

### Study Design and Patients

We included patients from our prospective registry of consecutive potentially thrombectomy‐eligible patients with acLVO treated from January 1, 2017 to January 1, 2023 at the tertiary stroke center of University Hospital Carl Gustav Carus in Dresden, Germany, in a retrospective cohort study. This study is reported in compliance with the Strengthening the Reporting of Observational Studies in Epidemiology statement.^11^ The Strengthening the Reporting of Observational Studies in Epidemiology checklist is shown in Data S1. We included only adults who had acute ischemic stroke due to imaging‐confirmed occlusion of the intracranial segment of the internal carotid artery (ICA) or the M1 or M2 segment of the middle cerebral artery with an established indication for thrombectomy. This encompassed those who have undergone thrombectomy with or without prior IVT as well as those for whom thrombectomy was omitted after performance of IVT, for example, because of early recanalization on repeated computed tomography (CT) angiography or angiogram. We excluded patients with posterior circulation stroke as well as those with unknown onset of symptoms and unknown time of last seen well, unknown baseline National Institute of Health Stroke Scale (NIHSS) score, or missing modified Rankin Scale (mRS) score at 90 days. Details on study selection criteria and reasons for omission of thrombectomy are provided in the study flowchart (Figure).

**Figure .**
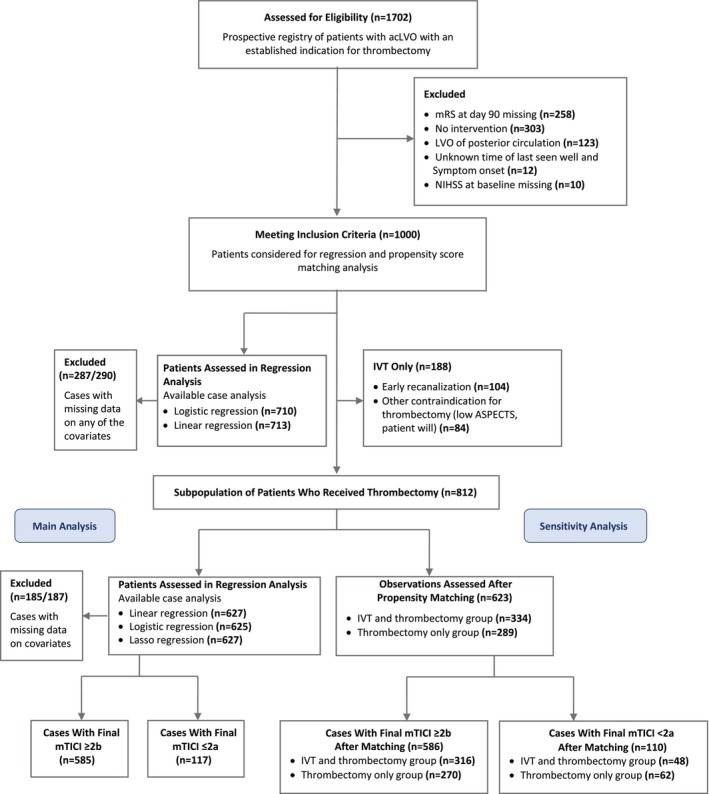
Study flowchart illustrating the results of the screening and selecting patients for inclusion in the main analysis as well as the sensitivity and subgroup analyses of the study. acLVO indicates anterior circulation large‐vessel occlusion; ASPECTS, Alberta Stroke Program Early Computed Tomography score; IVT, intravenous thrombolysis; LVO, large‐vessel occlusion; mRS, modified Rankin Scale; mTICI, modified Thrombolysis in Cerebral Infarction score; and NIHSS, National Institutes of Health Stroke Scale.

### Clinical and Imaging Assessment

Our thrombectomy registry encompasses both mothership patients and drip‐and‐ship transfers from 13 community hospitals without a neurology department that are spokes of our telestroke network or from our 8 partner hospitals who have neurological departments but no or limited thrombectomy capacity. Details of our regional stroke network have been published elsewhere.^12^ Our registry of thrombectomy‐eligible patients comprises detailed data on demographic characteristics, premorbid condition, chronic comorbidity, cardiovascular risk profiles, medication, stroke pathogenesis classified via TOAST (Trial of Org 10 172 in Acute Stroke Treatment), neurological deficits rated via NIHSS by stroke physicians and functional outcome via mRS scores at admission and discharge. Functional outcome was additionally obtained via a telephone interview 90 days after the day of intervention (IVT or thrombectomy), and favorable functional outcome was defined as an mRS score of 0 to 2 at the time of this follow‐up. Brain and vessel imaging findings included Alberta Stroke Program Early Computed Tomography Score (ASPECTS) and mTICI, occlusion site, and leptomeningeal collateral status. While mTICI was performed to quantify the grade of anterograde reperfusion of patent vasculature that supplies the target brain tissue after thrombus removal, the term *recanalization* is used henceforth to refer to the restoration of artery patency at the occlusion site.^13^ Treatment times were determined for onset‐to‐needle, onset‐to‐groin, onset‐to‐recanalization, needle‐to‐groin, needle‐to‐recanalization, and groin‐to‐recanalization intervals.

Parameters of interest to our study that were not available in our registry were extracted via chart review by 2 independent investigators (A.S., S.S.). A complete list of parameters and modes of their acquisition is provided in Table S1. We categorized vessel occlusion sites from proximal to distal into 6 groups as follows: (1) tandem occlusion (extracranial ICA occlusion or high‐grade stenosis preceding ipsilateral anterior circulation large‐vessel occlusion); (2) carotid‐T occlusion (coexistence of distal intracranial ICA occlusion and ipsilateral proximal M1 and A1 occlusion); (3) carotid‐L occlusion (occlusion of distal intracranial ICA and proximal M1 segment); (4) carotid‐I occlusion (isolated intracranial ICA occlusion); (5) isolated M1 occlusion; and (6) occlusion of M1‐M2 junction or isolated M2 occlusion. We defined tandem occlusion as extracranial ICA occlusion or high‐grade stenosis (≥70% NASCET [North American Symptomatic Carotid Endarterectomy Trial] stenosis), preceding ipsilateral acLVO. Distal thrombus migration was defined as change from a proximal to distal category on angiogram compared with preceding CT angiography or on repeated CT angiography, for example, following transfer from a drip‐and‐ship clinic to the mothership center. Distal thrombus migration beyond catheter accessibility resulting in omission of thrombectomy or complete absence of vessel occlusion on repeated CT angiography or angiogram was considered early recanalization. In these patients, time of recanalization was defined as time of the first imaging (CT angiography or angiogram) that did not show a sustained occlusion within catheter reach. Thrombectomy with successful reperfusion was defined as a postinterventional mTICI score of 2b or higher. In cases without catheter angiography, for example, because of early recanalization or insufficient core/penumbra mismatch on perfusion imaging at the mothership clinic following drip‐and‐ship transfer, mTICI scores were assessed using CT angiography post hoc by 2 experienced neuroradiologists (D.K., A.C.) as previously described.^14^ Consensus was reached for ambiguous findings. The sedative regimen during thrombectomy was classified as general anesthesia or conscious sedation. Further details on the definitions of patient characteristics are shown in Data S1.

### Ethical Standard

Our study was approved by the local institutional review board (Ethikkommission an der TU Dresden, institutional review board reference number: EK 272072017). Written informed consent for participation was waived in accordance with the national legislation and the institutional requirements.

### Statistical Analysis

For analysis, the study population was subdivided into 3 groups of patients receiving either IVT only, bridging IVT followed by thrombectomy, or thrombectomy only. Independent continuous variables were checked for normality using descriptive and analytic (Shapiro–Wilk test) criteria. Between‐group differences of demographic, clinical, imaging, and procedural characteristics were assessed using Fisher's exact test for binary data, the Kruskal–Wallis test for ordinal or nonnormally distributed continuous data, and 1‐way ANOVA for normally distributed data where appropriate.

We performed both linear and logistic regression in the entire study population as well as in the subpopulation of all patients who underwent thrombectomy to study functional outcome. Logistic regression was applied to assess the association of bridging IVT with favorable functional outcome defined as a mRS score of 0 to 2 at 90 days. Moreover, linear regression was performed to quantify the association of bridging IVT with mRS score when handled as continuous variable to capture smaller associations. In the subpopulations of patients with successful reperfusion and patients without successful reperfusion, we did not perform logistic regression because of the lower sample sizes of these subgroups with consequently weaker anticipated associations. Performance of IVT was treated as a binary variable. Covariates adjusted for were chosen by clinical reasoning and comprised age, premorbid dependency, chronic disease possibly impairing functional independence, malignancy, arterial hypertension, glycated hemoglobin (%), low‐density lipoprotein (mg/dL), smoking, stroke pathogenesis, NIHSS score at baseline, ASPECTS, vessel site, tandem occlusion, carotid‐T occlusion, mTICI score, emergency carotid stenting, thrombectomy, onset‐to‐recanalization time, and stroke pathogenesis as defined by TOAST category. Further definitions of covariates are detailed in Data S1. Residuals were tested for normality. Multicollinearity was assessed by calculating the variable inflation factor for all covariates in the regression model. A variable inflation factor value of 1 indicates no multicollinearity, whereas variable inflation factor values >5 indicate relevant multicollinearity. Where multicollinearity impaired interpretability of the regression model, double selection lasso linear regression for inference using cross validation and controlling for all covariates included in the original regression model was used to obtain reliable results. Interaction terms were included in regression models to assess independency of associations of IVT, distal thrombus migration, and final mTICI score with functional outcome after 90 days.

We conducted a sensitivity analysis using propensity score matching to test the robustness of the results on the average association of IVT with 90‐day functional outcome in the subpopulation of patients who underwent thrombectomy while additionally accounting for the nonrandomized study design. Each subject's propensity score was estimated by multivariable logistic regression incorporating the same covariates as used in the main model with the addition of a sedative regimen applied during thrombectomy. The maximum allowed difference in propensity scores for matching (caliper value) was targeted to be ≤0.2. Standardized differences and variance ratios were calculated to assess balance of covariates between the 2 groups of patients receiving either bridging IVT followed by thrombectomy or thrombectomy alone. We aimed for a standardized difference (mean±SD) of 0±0.1 and a variance ratio of 1±0.25. The aforementioned analyses were repeated in subgroups of patients who underwent thrombectomy with and without successful reperfusion. Significance level was set at α=0.05. Available case analysis was performed. The number of missing registry data is reported in Table S2 and was low. All analyses were performed using the statistical software package Stata (StataCorp, College Station, TX).

## Results

### Study Population

We included 1000 patients with acute ischemic stroke due to acLVO (513 women; median age, 77 years [interquartile range (IQR), 67–84 years]; baseline NIHSS score, 15 [IQR, 11–19]; baseline mRS score, 5 [IQR, 4–5]; median ASPECTS, 7 [IQR, 6–9]; median onset‐to‐needle time, 106 minutes [IQR, 81–135 minutes]; median onset‐to‐recanalization time 287 minutes [IQR, 219–358 minutes]). Of the 1000 patients, 402 (40.2%) received bridging IVT before thrombectomy, 188 (18.8%) received only IVT, and 410 (41.0%) underwent thrombectomy alone. The 90‐day mRS score was 4 (IQR, 1–6) in patients who received only IVT, 3 (IQR, 1–5) in those who received bridging IVT and subsequent thrombectomy, and 4 (IQR, 2–6) in those who underwent only thrombectomy. Of all patients who received IVT (n=590), 112 (18.9%) patients showed early recanalization. Distal thrombus migration was observed in 40 (6.7%) patients. Early recanalization occurred in 20 (12.6%) patients who received IVT following direct transportation to the mothership hospital of our telestroke network (n=159) versus 92 (21.4%) of those who received IVT at a drip‐and‐ship hospital (n=431). Distal thrombus migration occurred in 7 (4.4%) patients who received IVT following direct transportation to the mothership hospital of our telestroke network (n=159) versus 33 (7.6%) of those who received IVT at a drip‐and‐ship hospital (n=431). Demographic features, vascular risk profiles, and clinical and imaging characteristics are detailed in Table.

**Table .** Demographic and Baseline Characteristics

	IVT (n=188)	IVT + MT (n=402)	MT (n=410)	*P* value
Demographic characteristics				
Age, y, median, (IQR)	77 (68–84)	76 (6483)	79 (69–84)	0.013
Sex, female, n (%)	94 (49.5)	211 (52.8)	208 (50.7)	0.628
Premorbid condition				0.008
Independent, n (%)	135 (74.6)	310 (77.1)	277 (67.6)	
Needs assistance, n (%)	46 (25.1)	92 (22.9)	133 (32.4)	
Chronic disease, n (%)				0.076
No organ system	107 (58.8)	262 (65.3)	239 (58.3)	
1 Organ system	52 (28.6)	101 (25.2)	120 (29.3)	
2 Organ systems	20 (11.0)	35 (8.7)	45 (11.0)	
3 Organ systems	3 (1.7)	3 (0.8)	6 (1.5)	
4 Organ systems	…	…	…	
Malignancy, n (%)				0.0003
None	165 (87.7)	361 (89.8)	328 (80.0)	
1 Organ system	20 (10.6)	39 (9.7)	79 (19.3)	
2 Organ systems	3 (1.6)	2 (0.5)	2 (0.5)	
3 Organ systems	…	…	1 (0.2)	
Cardiovascular risk factors				
Arterial hypertension, n (%)	159 (88.3)	352 (88.0)	369 (90.0)	0.634
Diabetes, n (%)	54 (30.3)	112 (27.9)	120 (29.3)	0.810
Glycated hemoglobin, %, median (IQR)	5.7 (5.4–6.2)	5.8 (5.5–6.2)	5.8 (5.4–6.3)	0.910
Low‐density lipoprotein, mg/dL, mean (SD)	2.7±1.0	2.5 ±1.0	2.3±0.9	0.244
Preventive pharmacological treatment, n (%)				
Statin	56 (31.6)	123 (30.8)	141 (34.4)	0.530
Antiplatelet therapy	62 (35.6)	124 (31.4)	82 (20.1)	0.000
Stroke characteristics				
Wake‐up, n (%)	13 (7.0)	33 (8.2)	130 (32.9)	0.000
NIHSS at baseline, median (IQR)	16 (12–20)	16 (11–19)	15 (10–19)	0.026
NIHSS at discharge, median (IQR)	9 (2–42)	5 (1–15)	10 (3–19)	0.0001
mRS at baseline, median (IQR)	5 (4–5)	5 (4–5)	5 (4–5)	0.492
mRS at discharge, median (IQR)	4 (2–6)	3 (2–5)	4 (3–5)	0.0001
mRS at 90 d, median (IQR)	4 (1–6)	3 (1–5)	4 (2–6)	0.0001
mRS (0–2) at 90 d, n (%)	63 (33.5)	184 (45.8)	304 (74.2)	0.000
ASPECTS, median (IQR)	7 (4–9)	8 (6–9)	7 (6–9)	0.003
Occlusion side, left, n (%)	93 (49.5)	212 (52.8)	198 (48.3)	0.434
Occlusion site, n (%)				0.147
ICA intracranial	7 (3.7)	7 (1.7)	2 (0.5)	
L, M1 proximal‐distal	142 (75.5)	335 (83.3)	329 (80.2)	
M1/2‐transition, M2 proximal‐ distal	39 (20.7)	60 (14.9)	79 (19.3)	
Carotid‐T occlusion	24 (12.8)	36 (8.9)	44 (10.8)	0.352
Tandem occlusion	32 (17.2)	69 (17.2)	36 (8.8)	0.001
Leptomeningeal collaterals on DSA, n (%)	…	320 (81.0)	354 (86.6)	0.025
TOAST classification, n (%)				0.299
Large‐artery atherosclerosis	29 (16.1)	84 (20.9)	66 (16.2)	
Cardioembolism	104 (57.8)	229 (57.0)	247 (60.7)	
Small‐vessel occlusion	1 (0.6)	…	…	
Stroke of other determined pathogenesis	3 (1.7)	13 (3.2)	10 (2.5)	
Stroke of undetermined pathogenesis	43 (23.9)	76 (18.9)	84 (20.6)	
Interventions, n (%)				
Drip and ship	157 (83.5)	274 (68.2)	255 (62.2)	0.000
Carotid stent (emergency)	2 (1.1)	49 (12.2)	38 (9.3)	0.000
Sedative regimen, n (%)				0.430
Conscious sedation	…	115 (29.6)	117 (28.8)	
General anesthesia	…	273 (70.4)	289 (71.2)	
Procedural times, min, median (IQR)				
Onset unclear, n (%)	5 (2.7)	11 (2.7)	112 (27.3)	0.000
Onset‐to‐needle	108 (82–135)	105 (80–135)	…	0.560
Onset‐to‐groin	165 (130–210)	240 (165–285)	240 (175–313)	0.021
Onset‐to‐recanalization	210 (171–250)	302 (231.5–354.5)	296 (237–385)	0.0001
Groin‐to‐recanalization	…	52 (33–83)	53 (33–88)	…
Needle‐to‐groin	60 (42–129)	128 (60–160)	…	0.020
Procedural outcomes, n (%)				
Early recanalization	104 (55.3)	8 (2.0)	…	…
Distal thrombus migration	4 (2.1)	36 (9.0)	3 (0.7)	0.000
Thrombectomy frustrate	…	37 (9.4)	53 (13.0)	0.232
mTICI, n (%)				0.0001
0	69 (37.5)	30 (7.5)	42 (10.4)	
1	5 (2.7)	2 (0.5)	6 (1.5)	
2a	7 (3.8)	17 (4.2)	23 (5.6)	
2b	42 (22.8)	137 (34.1)	139 (33.9)	
2c, 3	61 (33.2)	216 (53.7)	200 (48.8)	
Successful reperfusion (mTICI ≥2b)	107 (56.9)	353 (87.8)	339 (82.7)	0.000

ASPECTS indicates Alberta Stroke Program Early Computed Tomography score; DSA, digital subtraction angiography; ICA, internal carotid artery; IQR, interquartile range; IVT, intravenous thrombolysis; mRS, modified Rankin Scale; MT, mechanical thrombectomy; mTICI, modified Thrombolysis in Cerebral Infarction score; NIHSS, National Institutes of Health Stroke Scale; and TOAST, Trial of Org 10 172 in Acute Stroke Treatment.

### Association of IVT and Favorable Functional Outcome in Thrombectomy‐Eligible Patients

In the entire study population, both logistic regression and linear regression substantiated a positive predictive association between performance of IVT and favorable functional outcome independent of grade of reperfusion quantified via mTICI and onset‐to‐recanalization time with additional adjustment for all predefined clinically relevant covariates (odds ratio, 0.49 [95% CI, 0.32–0.75]; *P*=0.001; ß=−0.36 [95% CI, −0.63 to −0.09]; *P*=0.01). This positive association remained significant on both logistic regression and linear regression when we included only patients who received thrombectomy with or without preceding bridging IVT (odds ratio, 0.49 [95% CI, 0.32–0.74]; *P*=0.001; ß=−0.39 [95% CI, −0.66 to −0.12]; *P*=0.01). In addition, ASPECTS was positively associated with favorable functional outcome, whereas negative predictive associations with favorable functional outcome were noted for premorbid dependency, presence of tandem occlusion, history of malignancy, higher baseline NIHSS score, age, higher glycated hemoglobin, and longer onset‐to‐recanalization time as detailed in Table S3.

We were able to confirm a positive independent association on sensitivity analysis using propensity score matching. Here, IVT was associated with an average 0.35‐point decrease in the mRS score at day 90 in patients who received bridging IVT compared with patients who received thrombectomy alone (ß=−0.35 [95% CI, −0.68 to −0.01]; *P*=0.04). The standardized differences and variance ratios are displayed in Table S4 and indicate a good match.

### Modulation of the Association of IVT and Functional Outcome by Grade of Reperfusion and Distal Thrombus Migration

We went on to assess whether the observed beneficial association of bridging IVT and functional outcome might be modulated by the interaction with grade of reperfusion. In the postestimation analysis, we observed multicollinearity for the association of bridging IVT and 90‐day functional outcome as well as the association of grade of reperfusion and 90‐day functional outcome possibly undermining a significant contribution of bridging IVT and mTICI score to 90‐day functional outcome (mean variable inflation factor, 19.8). To assess whether the association between IVT and 90‐day functional outcome differs with varying grades of reperfusion, we added an interaction term to the lasso regression for inference, factoring in the interaction between IVT and mTICI score. In this model, the interaction term remained nonsignificant as detailed in Table S5, whereas the main association of IVT and 90‐day functional outcome remained statistically significant (ß=−1.79 [95% CI, −3.43 to −0.15]; *P*=0.03). Higher mTICI scores were incrementally associated with improvement of 90‐day functional outcome (mTICI2a: ß=−0.85 [95% CI, −1.63 to −0.07]; *P*=0.03; mTICI 2b: ß=−1.57 [95% CI −2.19 to −0.96]; *P*=<0.001; mTICI 2c/3: ß=−1.79 [95% CI, −2.42 to −1.16]; *P*=<0.001) without compromising the independent beneficial association of IVT and 90‐day functional outcome. Bridging IVT was also associated with an improved 90‐day functional outcome when accounting for its interaction with distal thrombus migration (ß=−0.41 [95% CI, −0.69 to −0.12]; *P*=0.004). The interaction of the association of IVT and 90‐day functional outcome with distal thrombus migration was negative (ß=0.34 [95% CI, −1.7 to 0.49]; *P*=0.42).

### Association of Bridging IVT and Functional Outcome Following Thrombectomy With Successful Reperfusion

In due consideration of the aforementioned absence of an interaction between grade of reperfusion and the association of bridging IVT and functional outcome, we performed complementary subgroup analyses in patients who underwent thrombectomy with and without successful reperfusion defined as a final mTICI score of ≥2b and ≤2a, respectively. In patients with successful reperfusion, bridging IVT remained a positive predictor for improved 90‐day functional outcome on multivariable linear regression adjusted for age, sex, premorbid condition, baseline NIHSS score, ASPECTS, vessel occlusion site, thrombus migration, presence of tandem occlusion or carotid‐T occlusion, onset‐to‐recanalization time, carotid stenting, chronic disease, malignancy, arterial hypertension, glycated hemoglobin, low‐density lipoprotein, TOAST category, presence of leptomeningeal collaterals, and sedative regimen (ß=−0.45 [95% CI, −0.74 to −0.17]; *P*=0.002). A list of covariates associated with an improved 90‐day functional outcome is detailed in Table S6. We were able to reproduce the beneficial association of bridging IVT and 90‐day functional outcome in thrombectomy with successful reperfusion on propensity score matching analysis where bridging IVT was associated with an 0.50‐point decrease in final mRS score (ß=−0.50 [95% CI; −0.84 to −0.16]; *P*=0.004). The standardized differences and variance ratios are displayed in Table S7 and overall indicate a good match.

### Association of Bridging IVT and Functional Outcome in Thrombectomy With Unsuccessful Reperfusion

In the subgroup of thrombectomy patients who did not show successful reperfusion (final mTICI ≤2a), bridging IVT was still associated with improved 90‐day functional outcome on multivariable regression (ß=−0.47 [95% CI, −096 to 0.009]; *P*=0.05). A reduced number of clinically reasonable covariates was included in the multivariable linear regression model to avoid overfitting due to the lower sample size of this subgroup. The covariates were age, premorbid condition, baseline NIHSS score, ASPECTS, vessel occlusion site, presence of tandem occlusion, and TOAST category. Covariates significantly modifying functional outcome are detailed in Table S8.

Standardized differences and variance ratios of the sensitivity analysis using propensity matching are displayed in Table S9 and overall indicate a good match. Sensitivity analysis confirmed a beneficial association of bridging IVT before thrombectomy, with 90‐day functional outcome yielding a 0.37‐point decrease in 90‐day mRS score (ß=−0.37 [95% CI, −0.74 to −0.01]; *P*=0.05) when compared with thrombectomy alone.

## Discussion

The main finding of this study is that bridging IVT for anterior circulation stroke due to acLVO compared with thrombectomy alone improves functional outcome to an extent that cannot be explained solely by facilitated reperfusion as quantified by mTICI score.

In the light of inconclusive observations on the association of bridging IVT for acLVO and functional outcome from several RCTs and meta‐analyses,^3^, ^4^, ^5^, ^6^, ^7^, ^8^, ^9^, ^15^ recent research focused on deeper exploration of patient‐ and treatment‐related characteristics that might modulate this association. A retrospective analysis of the International Stroke Perfusion Imaging Registry (n=323) suggested that bridging IVT (n=241) is beneficial in patients with fast growing infarct core due to more rapid completion of thrombectomy resulting in a reduced final size of the ischemic lesion.^16^ In a prespecified secondary analysis of the DIRECT MT  (n=640), functional outcome was overall worse in more proximal compared with more distal occlusions, but the effect of bridging IVT (n=325) on functional outcome was not modulated by occlusion site when differentiating ICA, M1, and M2 occlusions.^17^ In post hoc analyses of the prospective observational studies Identifying New Approaches to Optimize Thrombus Characterization for Predicting Early Recanalization and Reperfusion With IV Alteplase and Other Treatments Using Serial CT Angiography (INTERRSeCT) and MR CLEAN registry, distal thrombus migration on repeated CT angiography or angiogram was associated with better functional outcome patients with acLVO.^18^, ^19^ In this regard, another post hoc analysis of the DIRECT MT trial found that distal thrombus migration resulting in an Expanded Thrombolysis in Cerebral Infarction score ≥2a before thrombectomy correlated with an improved functional outcome regardless of the final Expanded Thrombolysis in Cerebral Infarction score, leading the authors to conclude that the use of bridging IVT, which promotes early reperfusion, reduces ischemia time in the reperfused tissue and allows for blood flow through already recanalized collateral vessels.^20^ In this analysis, the beneficial effect of bridging IVT became apparent when thrombectomy got delayed more than half an hour. In our study, the observed net association of IVT and functional outcome was adjusted for onset‐to‐recanalization time to account for cases of early recanalization following IVT with subsequent omission of thrombectomy in the overall population of patients with acLVO as well as for procedure times in the subpopulation of patients who received thrombectomy. Furthermore, in our study, distal thrombus migration and early recanalization could be detected in 152 (25.8%) of the follow‐up CT angiographies or angiograms in patients who received IVT (n=590). The fraction was higher in patients who received IVT at the drip‐and‐ship hospital compared with the mothership clinic (29% versus 17.0%) likely due to the dilutive effects of longer exposure times to IVT as previously suggested by several studies and subgroup analyses of randomized trials.^17^, ^18^, ^19^, ^20^, ^21^ Nevertheless, the positive net association of bridging IVT and functional outcome was modulated neither by distal thrombus migration nor by the final mTICI score. Since these observations suggest that the success of bridging IVT does not depend solely on the success of subsequent thrombectomy, we repeated analysis in subgroups of patients who underwent thrombectomy with and without successful reperfusion. In both subgroups, we were able to confirm a positive net association of bridging IVT and improved functional outcome on repeated primary analysis as well as sensitivity analysis. Our observation in the subgroup of patients undergoing thrombectomy not achieving successful reperfusion interventionally is consistent with a recent cohort study (n=756) showing improved functional outcome in patients with acLVO with unsuccessful reperfusion following thrombectomy.^10^ Moreover, our observation of slightly improved functional outcome with an average 0.35‐point decrease in the mRS score at day 90 in patients who received bridging IVT compared with patients who received thrombectomy alone is consistent with a recent individual patient data meta‐analysis that has not been able to establish noninferiority of thrombectomy alone compared with IVT plus thrombectomy in patients presenting directly at thrombectomy centers.^22^

A continued beneficial effect of IVT beyond the physiologically momentous event of recanalization during thrombectomy might be explained by a sustained pharmacologic effect of recombinant tissue‐type plasminogen activator on the cerebral microcirculation. Congruently, in a transient middle cerebral artery occlusion rat model, recombinant tissue‐type plasminogen activator improved microvascular perfusion by reducing platelet aggregation in a fibrinogen‐dependent fashion with consequential reduction of downstream microvascular thrombosis.^23^ Translating this observation into human stroke survivors, a randomized placebo‐controlled clinical trial in 121 patients with acLVO found that intra‐arterial application of recombinant tissue‐type plasminogen activator in patients after thrombectomy with successful reperfusion increased the likelihood of achieving an excellent functional outcome at 90 days defined as an mRS score of 0 to 1.^24^

### Strengths and Limitations

Our observation of improved functional outcome following bridging IVT derived from a retrospective analysis of a prospective registry of thrombectomy‐eligible patients with partially imbalanced groups but showed high reproducibility on sensitivity analysis using propensity score matching and is independent of the grade of reperfusion. While the direction and significance of the assertions in our analyses are meaningful and unambiguous, the exact values of the continuous coefficients may be of limited absolute interpretability due to varying ranges of the different parameters by nature. Despite the goodness of fit of our regression models that is considered decent, still some amount of data remains unexplained by multivariable regression models. Real‐world big data analysis could help identify new parameters that modulate the association of bridging IVT and functional outcome while avoiding overfitting. A major reason why the question of whether bridging IVT before thrombectomy is beneficial has not yet been answered conclusively by observational or interventional research, including our data, might be that regression analysis with multiple covariates is used as a tacit predictive model for which studies have not been powered sufficiently. While our registry of patients with acLVO requiring thrombectomy is of a multicentric nature, encompassing a large telestroke network, thrombectomy was solely performed at the mothership clinic in our study. However, highly standardized acute and postinterventional stroke care as well as reproducibility of observations on propensity score–based sensitivity analyses and subgroup analyses support the internal validity and generalizability of our findings.

## Conclusions

Bridging IVT is beneficial with respect to functional outcome in patients with acLVO stroke who have an established indication for thrombectomy regardless of the achieved grade of reperfusion as quantified by the mTICI score and occurrence of distal thrombus migration.

## Sources of Funding

None.

## Disclosures

Dr Kaiser is supported by the Joachim Herz Foundation. Dr Siepmann received grants from the German Federal Ministry of Health and Kurt Goldstein Institut, speaker fees from AstraZeneca and honoraria from Dresden International University for serving as a program director and a lecturer of the Master's Program in Clinical Research at Dresden International University (DIU) as well as royalties from Thieme. This study was part of a Master's thesis at DIU. None of the other activities listed were related to the current study. The remaining authors have no disclosures to report.

## Supporting information

Data S1
Tables S1–S9
Reference 25
