# Supplementary material for: Intravenous Thrombolysis Before Thrombectomy Improves Functional Outcome After Stroke Independent of Reperfusion Grade
Source: J Am Heart Assoc. 2024 Mar 8;13(6):e031854. doi: 10.1161/JAHA.123.031854 (PMC11009998; doi:10.1161/JAHA.123.031854)
Supplement: Supplementary file 1 — Data S1 Tables S1–S9 Reference 25 [file JAH3-13-e031854-s001.pdf]

Supplemental Material

Data S1. Supplemental Methods

Definitions of Patient Characteristics

1. National Institutes of Health Stroke Scale at Baseline

Drip and ship patients from a community hospital without neurological expertise were evaluated via our telestroke network by a stroke fellow. Drip and ship patients transferred from a hospital with a neurological department as well as mothership patients were evaluated by local neurologists. All patients were evaluated for baseline NIHSS. In cases where the NIHSS score was missing but a detailed report of the neurological physical examination was available, NIHSS score was calculated by an investigator (AS) post hoc. Mechanical ventilation with sedation was set a score of 32 points on the NIHSS scale.

2. Premorbid State

Patients were classified to be dependent, when regular assessment indicated a permanent necessity of aid in any aspect of the activities of daily living.[25]

3. Stroke Etiology

Embolic stroke of undetermined source (ESUS) was subsumed under the TOAST category "stroke of undetermined etiology.

4. Chronic Disease

Diseases possibly reducing live expectancy or independency were included. Diseases were grouped to the following organ systems with 1 point for each system:

- Cor/Vascular: Coronary heart disease, high grade vitium, history of myocardial infarction, peripheral artery disease, history of carotid artery stent, noncompaction cardiomyopathy
- Lung: asthma, chronic obstructive pulmonary disease, pulmonary hypertension, chronic hypoventilation syndrome
- Renal: any renal insufficiency
- Abdomen: autoimmune and chronic hepatitis, chronic pancreatitis, sclerosing cholangitis, CCE, liver failure, Billroth I operation
- Other organ systems: Churg-Strauss syndrome, rheumatoid arthritis, neurodegenerative diseases, myasthenia gravis, nicht steatosis hepatis, neurosarcoidosis, critical illness polyneuropathy and myopathy, polymyalgia rheumatica, systemic sclerosis, Factor V Leiden, paranoid schizophrenia, infantile brain damage, muscular dystrophy, multiple sclerosis, morbid obesity

***Table S1**: List of Baseline and Study Parameters with Mode of Acquisition*

	**Available in prospective database**	**Extracted retrospectively via chart review**
Age, years (median [IQR])	X	
Sex, female (n, %)	X	
Premorbid condition		X
Chronic disease (n, %)		X
Cardiovascular risk factors		
Arterial hypertension (n, %)	X	
Diabetes mellitus (n, %)	X	
HbA1c, % (median [IQR])		X
LDL, mg/dl (mean, SD)		X
Nicotine (n, %)	X	
Malignancy (n, %)		X
Medication		
Statin (n, %)		X
Antiplatelet therapy (n, %)		X
Stroke characteristics		
Wake-up	X	
NIHSS at baseline (median, [IQR])	X	
NIHSS at discharge (median, [IQR])	X	
mRS at baseline (median [IQR])	X	
mRS at discharge (median [IQR])	X	
mRS at 90 days (median [IQR])	X	
mRS (0-2) at 90 days (n, %)	X	
ASPECTS (median [IQR])	X	
Occlusion side, left (n, %)	X	
Occlusion site (n, %)	X	
Carotid T occlusion (n, %)		X
Tandem occlusion (n, %)		X
Leptomeningeal collaterals on DSA (n, %)	X	
TOAST classification (n, %)		
Interventions (n, %)		
Drip and ship	X	
Carotid stent (emergency)	X	
Sedative regimen (n, %)	X	
Procedural times, min (median [IQR])	X	
Procedural outcomes (n, %)		
Early recanalization		X
Distal thrombus migration		X
Thrombectomy frustrate	X	
mTICI (n, %)	X	

IQR, interquartile range; SD standard deviation; LDL, low density lipoprotein; HbA1C, Haemoglobin A1c; NIHSS, National Institutes of Health Stroke Scale; mRS, modified Rankin scale; ASPECTS, Alberta Stroke Program Early CT score; TOAST, Trial of Org 10172 in Acute Stroke Treatment; IVT, intravenous thrombolysis; mTICI, modified treatment in cerebral infarction score.

Table S2: *Missing Data in the Prospective Registry of Thrombectomy-Eligible Patients*

Missing data (n, %)	IVT (n= 188)	IVT and MT (n=402)	MT (n=410)
Age	0, 0	0, 0	0, 0
Sex, female	0, 0	0, 0	0, 0
Arterial hypertension	8, 4.3	2, 0.5	0, 0
Diabetes mellitus	10, 5.3	0, 0	0, 0
Nicotine	5, 2.7	2, 0.5	0, 0
Wake-up stroke	1, 0.5	1, 0.3	15, 3.7
NIHSS at baseline	0, 0	0,0	0,0
NIHSS at discharge	5, 2.7	3, 0.8	2, 0.5
mRS at baseline	3,1.6	1,0.3	1, 0.3
mRS at discharge	6, 3.2	3, 0.8	2, 0.5
mRS at 90 days	0, 0	0, 0	0, 0
mRS (0-2) at 90 days	0, 0	0, 0	0, 0
ASPECTS	0, 0	3, 0.8	1, 0.2
Occlusion side, left	0, 0	0,0	0, 0
Occlusion site	0, 0	0,0	0, 0
Leptomeningeal collaterals on DSA	-	7, 1.7	1, 0.24
TOAST classification	8, 4.3	0, 0	3, 0.7
Drip and ship	0, 0	0, 0	0, 0
IVT (Extern, Intern)	0, 0	0, 0	0, 0
Carotid stent (emergency)	0, 0	0, 0	0, 0
Sedative regimen	-	14, 3.5	4, 1.0
Unclear symptom onset	5, 2.7	11, 2.7	112, 27.3
Onset-to-needle	2, 1.1	6, 1.5	-
Onset-to-groin	-	6, 1.5	52, 12.7
Onset-to-recanalization	90, 47.9	34, 8.5	91, 22.2
Groin-to-recanalization	-	35, 8.8	51, 12.4
Needle-to-groin	-	7, 1.7	-
Thrombectomy frustrate	-	9, 2.2	2, 0.5
mTICI	4, 2.1	10, 2.5	0, 0

NIHSS, National Institutes of Health Stroke Scale; mRS, modified Rankin scale; ASPECTS, Alberta Stroke Program Early CT score; DSA, digital subtraction angiography; TOAST, Trial of Org 10172 in Acute Stroke Treatment; IVT, intravenous thrombolysis; mTICI, modified treatment in cerebral infarction score.

Table S3: *Association of Bridging IVT and Functional Outcome in the Subpopulation of Thrombectomy-Eligible Patients*

Covariate	Association with functional outcome	Odds Ratio	95% Confidence interval	p-value
Increase in ASPECTS	beneficial	0.79	0.69-0.90	<0.001
Premorbid dependency	detrimental	3.50	2.07-5.92	<0.001
Tandem occlusion	detrimental	2.07	1.07-4.01	0.03
(History of) malignancy	detrimental	1.78	1.02-3.10	0.04
Increase in HbA1c	detrimental	1.41	1.10-1.80	0.01
Increase in NIHSS at baseline	detrimental	1.13	1.08-1.17	<0.001
Increase in age	detrimental	1.04	1.02-1.06	<0.001
Increase in onset-to-recanalization time	detrimental	1.002	1.001-.004	0.005

For multivariable linear regression $R^2 = 0.4$ indicated an acceptable goodness of fit of the regression model to the data. Post estimation calculation of variance inflation factors (VIF) did not indicate relevant multicollinearity (mean variance inflation factor = 4.2). IVT, intravenous thrombolysis; ASPECTS, Alberta Stroke Program Early CT score; DSA, digital subtraction angiography; HbA1C, Haemoglobin A1c; NIHSS, National Institutes of Health Stroke Scale.

***Table S4:** Covariates, Standardized Differences and Variance Ratios of Propensity Score Matching in Patients Receiving Thrombectomy*

Covariates	Standardized differences		Variance ratio	
	Raw	Matched	Raw	Matched
Age	-0.31	0.01	1.57	1.28
Sex	-0.07	0.01	1.00	1.00
Premorbid status	-0.22	-0.03	0.80	1.00
Chronic disease	-0.20	0.02	0.85	0.94
Malignancy	-0.19	0.07	0.63	1.20
NIHSS at baseline	0.11	0.02	1.00	0.91
Vessel site	-0.05	-0.13	1.06	1.02
Tandem occlusion	0.23	0.04	1.68	1.09
Carotid T occlusion	-0.06	-0.02	0.85	0.94
ASPECTS	0.08	-0.03	0.95	1.10
HbA1c (%)	0.05	-0.08	1.22	0.88
LDL (mg/dl)	0.21	0.01	1.25	1.00
Onset-to-recanalization time (min)	-0.23	0.06	0.41	0.92
Onset-to-recanalization time² (min²)	-0.28	0.03	0.25	0.92
Leptomeningeal collaterals	-0.14	-0.17	1.30	1.41
Sedation regimen	-0.02	-0.12	1.02	1.13
Final mTICI				
1	-0.01	0.00	0.87	1.00
2a	-0.78	-0.02	0.71	0.91
2b	-0.09	0.09	0.96	1.04
2c/3	0.11	-0.05	0.97	1.01
TOAST category				
1. Large artery atherosclerosis	Reference			
2. Cardioembolism	-0.08	-0.05	1.03	1.03
3. Small vessel occlusion	Empty			
4. Stroke of other determined etiology	0.02	-0.03	1.10	0.86
5. Stroke of undetermined etiology	-0.01	0.05	0.98	1.10

Each subject was matched to one nearest neighbor of the opposite group. The number of observations in the matched population was n=1246. Onset-to-recanalization time was included together with its quadratic term to successfully aim for a standardized difference of 0 ± 0.1 and a variance ratio of 1 ± 0.25 which could be achieved for all variables except age (variance ratio 1.28) and leptomeningeal collaterals (variance ratio 1.4). National Institutes of Health Stroke Scale; ASPECTS, Alberta Stroke Program Early CT score; Haemoglobin A1c; LDL, low density lipoprotein; mTICI, modified treatment in cerebral infarction score; TOAST, Trial of Org 10172 in Acute Stroke Treatment.

***Table S5:** Significances of Interaction Terms of IVT with Grades of Reperfusion*

mTICI Score	ß-coefficient	95% Confidence interval	p-value
0	Reference level		
1	1.58	-0.28-3.43	0.10
2a	1.66	-0.29-3.61	0.10
2b	1.61	-0.07-3.28	0.06
2c/3	1.24	-0.43-2.91	0.15

IVT, intravenous thrombolysis; mTICI, modified treatment in cerebral infarction score.

Table S6: *Association of Bridging IVT and Functional Outcome in Thrombectomy with Successful Reperfusion*

Covariate	Association with functional outcome	ß-coefficient	95% Confidence interval	p-value
Increase in ASPECTS	beneficial	-0.15	-0.24- -0.07	0.001
Nicotine	beneficial	-0.56	-1.03- -0.98	0.02
(History of) malignancy	detrimental	0.56	0.20-0.91	0.02
Increase in HbA1c	detrimental	0.33	0.16-0.49	<0.001
Increase in NIHSS at baseline	detrimental	0.08	0.06-0.11	<0.001
Increase in age	detrimental	0.03	0.01-0.04	<0.001

For multivariable linear regression R^2 = 0.4 indicated an acceptable goodness of fit of the regression model to the data. There was no multicollinearity (mean variance inflation factor 1.32). IVT, intravenous thrombolysis; ASPECTS, Alberta Stroke Program Early CT score; HbA1C, Haemoglobin A1c; NIHSS, National Institutes of Health Stroke Scale.

Table S7: *Propensity Matching Analysis of the Association of Bridging IVT and Functional Outcome following Thrombectomy with Successful Reperfusion - Covariance Balance Summary*

Covariates	Standardized differences		Variance ratio	
	Raw	Matched	Raw	Matched
Age	-3.17	0.02	1.51	1.23
Sex	-0.08	0.01	1.00	1.00
Premorbid status	-0.21	-0.03	0.80	1.03
Chronic disease	-0.21	0.06	0.88	1.12
Malignancy	-0.20	0.07	0.61	1.18
NIHSS at baseline	0.11	-0.02	1.00	1.04
Vessel site	-0.03	-0.004	1.11	1.23
Tandem occlusion	0.26	-0.03	1.86	0.94
Carotid T occlusion	-0.06	0.02	0.84	1.08
ASPECTS	0.09	0.01	0.95	0.98
HbA1c (%)	0.09	0.07	1.34	1.18
LDL (mg/dl)	0.21	0.03	1.23	1.13
Onset-to-recanalization time (min)	-.025	-0.09	0.38	0.80
Onset-to-recanalization time² (min²)	-0.30	-0.09	0.21	0.68
Leptomeningeal collaterals	-0.11	-0.02	1.26	1.04
Sedation regimen	-0.01	-0.03	1.01	1.03
TOAST category	-0.06	-0.09	1.04	0.95
1. Large artery atherosclerosis	Reference			
2. Cardioembolism	-0.10	-0.06	1.04	1.02
3. Small vessel occlusion	0 observations			
4. Stroke of other determined etiology	-0.003	0.005	0.98	1.03
5. Stroke of undetermined etiology	-0.01	0.004	0.98	1.01

Each subject was matched to two nearest neighbors of the opposite group. The number of observations in the matched population was n= 1172. IVT, intravenous thrombolysis; NIHSS, National Institutes of Health Stroke Scale; ASPECTS, Alberta Stroke Program Early CT score; Haemoglobin A1c; LDL, low density lipoprotein; mTICI, modified treatment in cerebral infarction score; TOAST, Trial of Org 10172 in Acute Stroke Treatment.

Table S8: *Association of Bridging IVT and Functional Outcome Following Thrombectomy with Unsuccessful Reperfusion*

Covariate	Association with functional outcome	ß-coefficient	95% Confidence interval	p-value
Increase in NIHSS at baseline	detrimental	0.10	0.02-0.11	0.01
Increase in age	detrimental	0.03	0.001-0.054	0.05

R^2 = 0.3. There was no relevant multicollinearity (mean variance inflation factor = 1.40). IVT, intravenous thrombolysis; NIHSS, National Institutes of Health Stroke Scale.

Table S9: *Propensity Matching Analysis of the Association of Bridging IVT and Functional Outcome following Thrombectomy with Unsuccessful Reperfusion - Covariance Balance Summary*

	Standardized differences		Variance ratio	
	Raw	Matched	Raw	Matched
Age	0.13	0.06	1.16	1.24
Sex	0.14	-0.09	0.97	0.99
Premorbid status	-0.05	-0.16	0.96	0.87
NIHSS at baseline	0.17	-0.11	0.87	0.87
Vessel site	-0.41	-0.02	0.60	1.10
Tandem occlusion	0.25	0.00	1.61	1.00
ASPECTS	-0.08	-0.07	0.84	1.23
$ASPECTS^2$	-0.10	-0.05	0.81	1.12
HbA1c (%)	-0.22	-0.05	0.85	1.30
TOAST category				
1. Large artery atherosclerosis	Reference			
2. Cardioembolism	0.06	0.10	0.99	0.97
3. Small vessel occlusion	0 observations			
4. Stroke of other determined etiology	0 observations			
5. Stroke of undetermined etiology	-0.07	-0.07	0.91	0.88

Each subject was matched to one nearest neighbor of the opposite group. The number of observations in the matched population was n=220. ASPECTS was included with an additional quadratic term to successfully aim for a standardized difference of 0±0.1 and a variance ratio of 1±0.25 which could be achieved for all variables except age (variance ratio 1.4) and HbA1c (variance ratio 1.3)

STROBE Statement—checklist of items that should be included in reports of observational studies

	Item No.	Recommendation	Page No.	Relevant text from manuscript
**Title and abstract**	1	(*a*) Indicate the study's design with a commonly used term in the title or the abstract	1-3	"retrospective cohort study"
		(*b*) Provide in the abstract an informative and balanced summary of what was done and what was found		"In thrombectomy-eligible acLVO patients IVT improved functional outcome independent of grade of reperfusion and distal thrombus migration."
**Introduction**				
Background/rationale	2	Explain the scientific background and rationale for the investigation being reported	4	"Several randomized controlled trials tested whether bridging IVT prior to thrombectomy has an additive beneficial effect on clinical outcome. These studies yielded conflicting results."
Objectives	3	State specific objectives, including any prespecified hypotheses	4-5	"to assess if IVT in thrombectomy-eligible acLVO patients has a beneficial effect on functional outcome beyond an extent that can be explained by improvement of reperfusion as captured by modified Thrombolysis in Cerebral Infarction (mTICI) scale"
**Methods**				
Study design	4	Present key elements of study design early in the paper	5	"…prospective registry…into a retrospective cohort study."
Setting	5	Describe the setting, locations, and relevant dates, including periods of recruitment, exposure, follow-up, and data collection	5-6	"treated from 01/01/2017 to 01/01/2023 at the tertiary stroke center of University Hospital Carl Gustav Carus in Dresden, Germany"
Participants	6	(*a*) *Cohort study*—Give the eligibility criteria, and the sources and methods of selection of participants. Describe methods of follow-up		"adults who had acute ischemic stroke due to imaging-confirmed defined as occlusion of the intracranial segment of the internal carotid artery (ICA) or the M1 and/or M2 segment of the middle cerebral

				artery (MCA) with an established indication for thrombectomy”
		(*b*) *Cohort study*—For matched studies, give matching criteria and number of exposed and unexposed<br>*Case-control study*—For matched studies, give matching criteria and the number of controls per case	9, Tables.S4 and S7	“The maximum allowed difference in propensity scores for matching (caliper value) was targeted to be <0.2. Standardized differences and variance ratios were calculated to assess balance of covariates between the two groups of patients receiving either bridging IVT followed by thrombectomy or thrombectomy alone.”
Variables	7	Clearly define all outcomes, exposures, predictors, potential confounders, and effect modifiers. Give diagnostic criteria, if applicable	8-9, Supplemental Methods	“Covariates adjusted for where chosen by clinical reasoning and comprised age, premorbid dependency, chronic disease possibly impairing functional independence, malignancy, arterial hypertension, HbA1c (%), low-density lipoprotein (LDL, mg/dl), smoking, stroke etiology, NIHSS at baseline, Alberta Stroke Program Early CT score (ASPECTS), vessel site, tandem occlusion, carotid T occlusion, mTICI score, emergency carotid stenting, thrombectomy, onset-to-recanalization time, and stroke etiology as defined by TOAST category.”
Data sources/ measurement	8*	For each variable of interest, give sources of data and details of methods of assessment (measurement). Describe comparability of assessment methods if there is more than one group	6-8, Table S1, Supplemental Methods	Parameters of interest to our study that were not available in our registry were extracted via chart review by two independent investigators (AS, SS). A complete list of parameters and modes of their acquisition is provided in Table S1.
Bias	9	Describe any efforts to address potential sources of bias	16-17	“..highly standardized acute and post-interventional stroke care as well as reproducibility of observations on propensity score-based sensitivity analyses and subgroup analyses support the internal validity and generalizability of our findings.”
Study size	10	Explain how the study size was arrived at	5, Figure 1	“We included patients from our prospective registry of consecutive potentially thrombectomy-eligible

				acLVO patients treated from XX-XX at the tertiary stroke center of University Hospital Carl Gustav Carus in Dresden, Germany into a retrospective cohort study.”
Quantitative variables	11	Explain how quantitative variables were handled in the analyses. If applicable, describe which groupings were chosen and why	8-10	“For analysis the study population was subdivided into three groups of patients receiving either IVT only, bridging IVT followed by thrombectomy or thrombectomy only.”
Statistical methods	12	(*a*) Describe all statistical methods, including those used to control for confounding	8-10	“Fisher’s exact test for binary data, Kruskal-Wallis test for categorical or non-normally distributed continuous data and one-way analysis of variance for normally distributed data where appropriate.” “We performed multivariable regression…” “Multicollinearity was assessed by calculating the variable inflation factor…” “…double selection lasso linear regression for inference using cross-validation and controlling for all covariates included in the original regression model…” “… propensity score matching…”
		(*b*) Describe any methods used to examine subgroups and interactions	9-10	“…in the subpopulation of patients that underwent thrombectomy while additionally accounting for the non-randomized study design.” “The aforementioned analyses were repeated analysis in subgroups of patients who underwent thrombectomy with and without successful reperfusion to further explore the effect of bridging IVT on functional outcome beyond its impact on thrombectomy-mediated recanalization.” “Interaction terms were included in regression models to assess independency of effects of IVT, distal thrombus migration and final mTICI score on functional outcome after 90

				days.”
		(*c*) Explain how missing data were addressed	10	“Available case analysis was performed.”
		(*d*) *Cohort study*—If applicable, explain how loss to follow-up was addressed	N/A	
		(*e*) Describe any sensitivity analyses	9	“We conducted a sensitivity analysis using propensity score matching to test the robustness of the results on the average effect of IVT on 90-day functional outcome in the subpopulation of patients that underwent thrombectomy while additionally accounting for the non-randomized study design.”
**Results**				
Participants	13*	(a) Report numbers of individuals at each stage of study—e.g. numbers potentially eligible, examined for eligibility, confirmed eligible, included in the study, completing follow-up, and analysed	5, Figure 1	“Details on study selection criteria and reasons for ommitance of thrombectomy are provided in the study flowchart (Figure 1).”
		(b) Give reasons for non-participation at each stage	Figure 1	see figure 1
		(c) Consider use of a flow diagram	Figure 1	see figure 1
Descriptive data	14*	(a) Give characteristics of study participants (eg demographic, clinical, social) and information on exposures and potential confounders	10-11, Table 1	“Demographic features, vascular risk profiles as well as clinical and imaging characteristics are detailed in Table 1.”
		(b) Indicate number of participants with missing data for each variable of interest	10, Table S2	“The number of missing registry data is reported in Table S2 and was low.”
		(c) *Cohort study*—Summarise follow-up time (eg, average and total amount)	Figure 1	see figure 1
Outcome data	15*	*Cohort study*—Report numbers of outcome events or summary measures over time	10-14, Table 1	“Ninety-day mRS was 4 [IQR, 1;6] in patients who received only IVT, 3 [IQR, 1;5] in those who received bridging IVT and subsequent thrombectomy and 4 [IQR, 2;6] those who underwent only thrombectomy..”
Main results	16	(*a*) Give unadjusted estimates and, if applicable, confounder-adjusted estimates and their precision (eg, 95% confidence interval). Make clear which confounders were adjusted for and why they were included	10-14	“In the entire study population multivariable linear regression substantiated a positive predictive association between performance of IVT and favorable functional outcome independent of grade of reperfusion quantified via mTICI and onset-to-recanalization time with additional adjustment for all predefined clinically relevant covariates (OR 0.49; 95% CI [0.32;0.75]; p=0.001).”

		(*b*) Report category boundaries when continuous variables were categorized		N/A
		(*c*) If relevant, consider translating estimates of relative risk into absolute risk for a meaningful time period		N/A
Other analyses	17	Report other analyses done—eg analyses of subgroups and interactions, and sensitivity analyses	10-14	"We were able to confirm a positive independent association on sensitivity analysis using propensity score matching."<br><br>"In patients with successful reperfusion bridging IVT remained a positive predictor for improved 90-day functional outcome on multivariable linear regression…"<br><br>"In the subgroup of thrombectomy patients who did not show successful reperfusion (final mTICI [illegible] 2a) bridging IVT was still associated with improved 90-day functional outcome on multivariable regression (ß=-0.47; 95% CI [-096;0.009]; p=0.05)."
**Discussion**				
Key results	18	Summarise key results with reference to study objectives	14	"The main finding of this study is that bridging IVT for anterior circulation stroke due to acLVO compared to thrombectomy alone improves functional outcome to an extent that cannot be explained solely by facilitated reperfusion as quantified by mTICI score."
Limitations	19	Discuss limitations of the study, taking into account sources of potential bias or imprecision. Discuss both direction and magnitude of any potential bias	16-17	Our observation of improved functional outcome following bridging IVT derived from a retrospective analysis of a prospective registry of thrombectomy-eligible patients with partially imbalanced groups but showed high reproducibility on sensitivity analysis using propensity score matching and is independent of the grade of reperfusion.
Interpretation	20	Give a cautious overall interpretation of results considering objectives, limitations, multiplicity of	14-16	"A continued beneficial effect of IVT beyond the physiologically momentous

		analyses, results from similar studies, and other relevant evidence		event of recanalization during thrombectomy might be explained by a sustained pharmacologic effect of recombinant tissue-type plasminogen activator (rtPA) on the cerebral microcirculation.”
Generalisability	21	Discuss the generalisability (external validity) of the study results	17	“While our registry of acLVO patients requiring thrombectomy is of multicentric nature, encompassing a large telestroke network, thrombectomy was solely performed at the mothership clinic. However, highly standardized acute and post-interventional stroke care as well as reproducibility of observations on propensity score-based sensitivity analyses and subgroup analyses support the internal validity and generalizability of our findings.”
**Other information**				
Funding	22	Give the source of funding and the role of the funders for the present study and, if applicable, for the original study on which the present article is based	17	“This study received no external funding.”

*Give information separately for cases and controls in case-control studies and, if applicable, for exposed and unexposed groups in cohort and cross-sectional studies.

Note: An Explanation and Elaboration article discusses each checklist item and gives methodological background and published examples of transparent reporting. The STROBE checklist is best used in conjunction with this article (freely available on the Web sites of PLoS Medicine at http://www.plosmedicine.org/, Annals of Internal Medicine at http://www.annals.org/, and Epidemiology at http://www.epidem.com/). Information on the STROBE Initiative is available at www.strobe-statement.org.
